# The association between oral health literacy and oral health behaviors in North Korean defectors: a cross-sectional study

**DOI:** 10.1186/s12889-020-08550-7

**Published:** 2020-07-07

**Authors:** Han-A Cho, Ae-Jung Im, Yu-Ri Sim, Han-Byoel Jang, Hee-Jung Lim

**Affiliations:** 1 Department of Dental Hygiene, Shinhan University, 95 Hoam-ro, Uijeongbu-si, Gyeonggi-do 11644 Republic of Korea; 2 Department of Dental Hygiene, Hyejeon College, 25 Daehak-gil (Rd). Hongseong-eup, Hongseong-gun, Chungcheongnam-do 32244 Republic of Korea; 3 Department of Dental Hygiene, Graduate School of Public Health Science, Eulji University, 553 Sanseong-daero, Sujeong-gu, Seongnam, 13135 Republic of Korea

**Keywords:** North Korean defector, Oral health behavior, Oral health literacy, Health education

## Abstract

**Background:**

The present study aimed to investigate the association between oral health literacy and oral health behaviors among North Korean defectors.

**Methods:**

This study involved the collection of self-reported questionnaires from 123 North Korean defectors visited a dental clinic that offered complimentary services, to receive dental treatment in a metropolitan area of South Korea from December 2017 to April 2018. Oral health literacy was measured with the Test of Korean Functional Health Literacy in Dentistry (TOKFHLiD), which consists of 30 items concerning verbal oral health literacy and 42 items concerning functional oral health literacy (28 items for reading comprehension and 14 items for numeracy). In addition, the questionnaire contains 15 and 14 items related to demographic characteristics and oral health behaviors (interest, lifestyle, diet, prevention), respectively, for a total of 101 items.

**Results:**

The mean oral health literacy score was 44 (out of a maximum possible score of 72). Oral health literacy and oral health behaviors were positively correlated (*r* = 0.526, *P* < 0.001), and oral health literacy also had a significant effect on oral health behaviors (Beta = 0.26, 95% CI: 0.04–0.33). However, although functional oral health literacy had a significant effect on oral health behaviors (Beta = 0.20, 95% CI: 0.01–0.43), verbal oral health literacy did not (Beta = 0.13, 95% CI: − 0.06-037).

**Conclusions:**

Educational interventions are needed to improve oral health literacy, and thus oral health behaviors, as a part of the health promotion measures undertaken to facilitate the stable adjustment of North Korean defectors in South Korean society.

## Background

From their first arrivals in the 1990s, the North Korean defector population in South Korea grew to approximately 10,000 in 2007, and to 31,827 defectors as of August 2018 [1]. The South Korean government enacted the North Korean Refugees Protection and Settlement Support Act in 1997 and has continuously implemented various policies to promote the stable settlement of North Korean defectors in South Korea. Per the Medical Care Assistance Act of the National Basic Living Security Act, North Korean defectors are provided healthcare benefits and are subject to reduced medical costs in local public hospitals [2, 3]. Furthermore, the Korea Hana Foundation provides treatment for chronic major conditions and general diseases, traditional Korean medical treatment, and dental treatment (e.g., complete dentures) [3]. The Hana Clinic of Hanawon, a settlement support center for North Korean defectors, provides primary care (internal medicine, traditional Korean medicine, dentistry, urology, obstetrics and gynecology, pediatrics, neurology), as well as secondary and tertiary care via partnerships with hospitals and volunteers [4]. Nevertheless, North Korean defectors still face difficulties in using health services due to a lack of relevant support; therefore, meeting their medical needs should be considered a priority to promote their adjustment to life in South Korea [5].

The total number of cases of care provided by the Hana Clinic to North Korean defectors from 1999 to 2016 was 333,626, with dental care comprising approximately 20% of the cases (2nd most frequently provided type of care) [4]. The number of people requiring treatment for periodontal disease (code 3), based on the Community Periodontal Index of Treatment Needs, has been reported to be higher among the North Korean defector population [6]. Among female North Korean defectors, approximately 81% are currently in need of dental treatment, but only 28% were reported to have undergone dental treatment [7]. Some factors that hinder the use of healthcare services include difficulties with “understanding doctor’s or nurses’ explanation,” “communicating opinions to the doctor during consultation about a disease,” “care procedure,” and “taking drugs or getting treatment.” This calls for special attention to the health management of this vulnerable part of the population [8]. Although South Koreans and North Koreans use a similar language, communication difficulties caused by differences in cultural background constitute the greatest difficulty for North Koreans in their use of medical services. This indirectly suggests that there may be a barrier hindering the use of dental health services and the acquisition of oral health information. Therefore, identifying these difficulties using instruments for measuring oral health literacy may provide data useful for the promotion of oral health in North Korean defectors.

Oral health literacy refers to the ability to search for, understand, and use relevant information amid a sea of oral health-related information [9], and it is assessed using some of the most commonly used oral health literacy assessment scales, such as the two versions of the Rapid Estimate of Adult Literacy in Dentistry, REALD-30 [10] and REALD-99 [11]; the Test of Functional Health Literacy in Dentistry (TOFHLiD) [12]; and the Oral Health Literacy Instrument (OHLI) [10], and its Korean version (OHLI-K) [13]. Most previous studies regarding the health of North Korean defectors were focused on the association between mental health and physical health [14], while other studies examined chronic diseases [15] and infectious diseases [16]. In contrast, there have been few studies regarding oral health and literacy. These have primarily investigated factors associated with oral health literacy in adults [17], oral health literacy of mothers in multicultural families [18], and the development of tools for assessing oral health literacy [13]. To date, no studies have examined the relationship between oral health literacy and oral health behaviors in North Korean defectors. It is important to accurately understand and discuss oral health and oral health literacy, since such a multi-perspective study has the potential to address the health-related needs of North Korean defectors, who are increasing as vulnerable groups. Elucidation of the relationship between these two measures of oral health would help to address the oral health-related needs of North Korean defectors.

Thus, the objective of the present study was to investigate the level of oral health literacy among North Korean defectors and its association with oral health behaviors. Based on these findings, we aimed to provide foundational data for the development of customized education programs, tailored to the verbal comprehension level of North Korean defectors, and implement relevant strategies in order to promote their adjustment to South Korean society.

## Methods

### Study design and participants

This study was a cross-sectional survey of self-reported questionnaires completed by North Korean defectors. It was conducted from December 2017 to April 2018 at a dental clinic that provided complimentary dental services to North Korean defectors in a metropolitan area of South Korea (Seongnam, Gyeonggi-do). Eligibility was limited to adults who received at least one treatment at this dental clinic. Among a total of 132 questionnaires, 9 with incomplete responses were excluded, resulting in a total of 123 questionnaires included in the final analysis. In accordance with the Declaration of Helsinki, the Institutional Review Board of Eulji University reviewed and approved the study protocol, including the consent procedure (IRB-2018-5).

### Questionnaire items and structure

The association between oral health literacy and oral health behaviors among North Korean defectors was measured using a modified and adapted version of the questionnaire used by Kim [13] and Lee [19]. The tools used have been confirmed for reliability and validity [20].

Oral health literacy was divided into verbal oral health literacy and functional oral health literacy, and the latter was further divided into reading comprehension and numeracy. Therefore, the functional values of oral health literacy could be evaluated by summing up the values of reading comprehension and numeracy. The values of functional oral health literacy and verbal oral health literacy were combined to determine the level of oral health literacy.

The questionnaire consisted of 30 items concerning verbal oral health literacy and 42 items concerning functional oral health literacy (28 and 14 items for reading comprehension and numeracy, respectively). In addition, it contained items related to demographic characteristics (15 items) and oral health behaviors (14 items), for a total of 101 items.

Oral health literacy was defined with reference to the Test of Korean Functional Health Literacy in Dentistry (TOKFHLiD), developed by Kim [13] with reference to the REALD and the TOFHLiD, questionnaires developed in the United States.

Verbal oral health literacy, with scores ranging from 0 to 30 points, was measured by dividing participants into two levels depending on whether they did or did not accurately understand 30 terms pertaining to oral health and disease, “enough to be able to describe them to other people” [13]. The 30 terms were: toothbrush, dental cavity (dental caries), periodontium (gum), smoking, sugar, denture, orthodontic appliance, teeth grinding, analgesics, dental floss, halitosis (bad breath), congestion, tooth extraction, early-stage, fluoride, sealant, malocclusion, genetic, dental state, dental plaque, temporomandibular joint, dental pulp (tooth nerve), abscess (pus), gingiva, cellulitis, restoration, hypoplasia, enamel, fistula, and apicoectomy. The higher the combined score, the higher the level of verbal oral health literacy.

Functional oral health literacy consisted of two sections related to reading comprehension and numeracy. Reading comprehension was measured as the selection of the appropriate word out of four choices to fill in blank spaces in various contexts: A treatment consent form (5 items), precautions for using sedatives (10 items), toothpaste product labels (2 items), toothbrush product labels (2 items), dental floss product labels (5 items), and interdental brush product labels (4 items). The numeracy section was used to evaluate the ability to understand instructions and was measured based on the comprehension of toothpaste product specifications (1 item), mouthwash product specifications (3 items), a dental appointment card (6 items), and instructions for a place to visit after treatment (4 items). A score of 1 point was given for a correct answer and 0 for an incorrect answer. The higher the combined score of reading comprehension and numeracy, the higher the level of functional oral literacy. Functional oral literacy ranged from 0 to 42 points, and total oral health literacy ranged from 0 to 72 points.

As general characteristics, we examined sex (male, female), age (19–39, 40–59, 60–80), level of education (primary school or lower, middle school, high school, college and higher), average monthly income (≤ 99, 100–199, ≥ 200 [Units: 10,000 KRW]), occupation (yes, no), marital status (single, married), years of living in Korea (< 7 years, ≥ 7 years), number of diseases diagnosed by a physician (0, 1, 2, ≥ 3), private insurance (yes, no), perceived oral health status (good, normal, poor), interest in perceived oral health status (good, normal, poor), frequency of toothbrushes per day (1, 2, ≥ 3), number of oral hygiene products used (1, 2, ≥ 3), and dental care utilization over the last year (yes, no). Oral health behaviors were measured using a questionnaire based on the tool used by Lee [19]. The questionnaire consisted of 14 items, with 2 items related to areas of interest, 5 to lifestyle habits, 4 to diet, and 3 to prevention. Each item was rated on a 5-point Likert scale, with 5 corresponding to “absolutely agree” and 1 to “absolutely disagree.” Higher scores indicated higher levels of oral health behavior, with a range of 14 to 70 points. The internal consistency (Cronbach’s α) of the questionnaire was 0.944, representing high reliability.

### Statistical analysis

The general characteristics of the participants are presented with descriptive statistics. Considering that the maximum total score is different for the two domains of oral health literacy, the mean scores were converted to a 100-point scale for direct comparison. In addition, the correlations between oral health literacy and oral health behaviors were analyzed with Pearson’s correlation test. The association between oral health literacy and oral health behaviors were analyzed with multiple linear regression models. In this case, the normality of the residuals was confirmed through kernel density estimation and residual plot (for confirmation of homoscedasticity). In addition, the correlation between verbal oral health literacy and functional oral health literacy was confirmed. Since high correlation coefficients can cause multicollinearity between independent variables, we examined variation inflation factors and tolerances. The coefficient estimated by the conditional number was 8.38, indicating that it was not at a level that affected multicollinearity (Table 1). Since North Korean defectors have poor oral health but have difficulty in accessing dental treatment owing to economic problems [7, 8], the socioeconomic characteristics of the subjects and oral health-related variables are included in the model. At this time, the covariates also confirmed multicollinearity through VIF.

**Table 1:** VIF and tolerance on Verbal oral health literacy and Functional oral health literacy

Variable	VIF	Tolerance	
Verbal oral health literacy, Functional oral health literacy	1.12	1.06	
Condition number			8.38

*VIF* Variance inflation factor

G-power 3.1.9.4 (Kiel University, Kiel, Germany) was used to perform the sample size calculation. The total sample size was determined to be 122, based on an error probability (significance level) of 0.05, and a power (1-*β*) of 0.78. We also set up 2 multivariable linear regression models to identify the influence of each explanatory factor on oral health behavior. The independent variables were set to be verbal oral health literacy and functional oral health literacy for model 1 and oral health literacy (verbal oral health literacy + functional oral health literacy) for model 2, with adjustment for demographic, general health, and oral health characteristics. The trends of oral health behaviors according to oral health literacy were plotted. Statistical significance was defined as *P*-value < 0.05. There were no missing values for any available variables. Statistical analyses were performed using Stata Version 14.0 MP (StataCorp LP., College Station, TX, USA).

## Results

### Demographics and oral health-related characteristics of the participants

The characteristics of the participants are presented in Table 2.

**Table 2:** Demographic and oral health-related characteristics of North Korean defectors in South Korea (N = No. of participants, % = percent)

	Variables	Categories	*N* = 123	%
Demographics	Sex	Male	42	34.2
		Female	81	65.8
	Age	19–39	45	36.6
		40–59	62	50.4
		60–80	16	13.0
	Education	≤ Primary school	19	15.5
		Middle school	22	17.9
		High school	58	47.1
		≥ College	24	19.5
	Income [Units: 10,000 KRW]	≤ 99	66	53.7
		100–199	40	32.5
		≥ 200	17	13.8
	Occupation	Yes	66	53.7
		No	57	46.3
	Marital status	Single	45	36.6
		Married	78	63.4
	Years living in South Korea	< 7	58	47.2
		≥ 7	65	52.8
	Number of diseases	0	42	34.1
		1	38	30.9
		2	19	15.5
		≥ 3	24	19.5
	Private insurance	Yes	50	40.7
		No	73	59.3
Oral health	Perceived oral health status	Good	14	11.4
		Normal	49	39.8
		Poor	60	47.8
	Interest in perceived oral health status	Good	60	48.8
		Normal	43	35.0
		Poor	20	16.2
	Tooth brushing	1	15	12.2
		2	42	34.2
		≥ 3	66	53.6
	Use of dental products	1	10	8.1
		2	64	52.0
		≥ 3	49	39.9
	Dental care utilization	Yes	63	51.2
		No	60	48.8

*KRW* South Korean won

### Oral health literacy and oral health behaviors in North Korean defectors

#### Mean oral health literacy scores

North Korean defectors scored higher in functional oral health literacy than in verbal oral health literacy. The mean verbal oral health literacy score was approximately 14 out of a maximum possible score of 30 (corresponding to a score of 47 on a 100-point scale), and the mean functional oral health literacy score was approximately 30 out of a maximum of 42 (71 on a 100-point scale). The total oral health literacy score (i.e., the sum of the verbal and functional oral health literacy scores) was approximately 44 (61 on a 100-point scale, Table 3).

**Table 3:** Mean oral health literacy scores of North Korean defectors

Oral health literacy score	Mean ± SD	Score on a 100-point scale
^a^Verbal oral health literacy	14.10 ± 7.79	46.95 ± 25.96
^b^Functional oral health literacy	29.81 ± 8.77	70.95 ± 20.87
Reading comprehension	22.18 ± 5.88	79.18 ± 21.01
Numeracy	7.63 ± 4.14	54.53 ± 29.61
Total	43.91 ± 13.50	61.04 ± 18.77

*SD* standard deviation

^a^ Reported from a maximum of 30 points, with 1 point awarded for each correct answer

^b^ Reported from a maximum of 42 points, with 1 point awarded for each correct answer

#### Trends of oral health behaviors according to oral health literacy

**Fig. 1:**
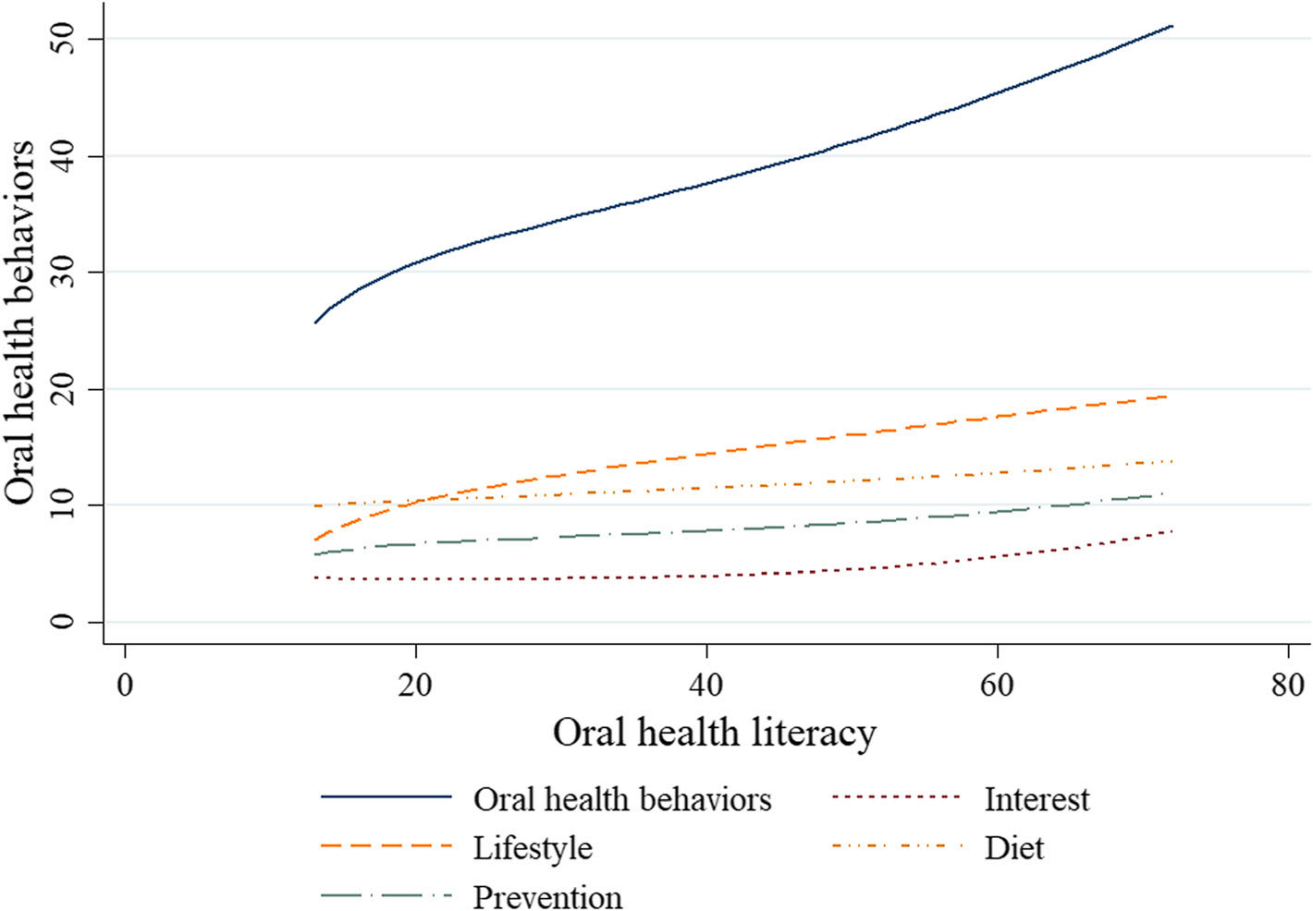
Trends of oral health behaviors according to oral health literacy in North Korean defectors

Figure 1 shows how oral health behaviors (70 points) of the Y-axis change as oral health literacy (72 points) of the X-axis increases. Oral health behaviors are expressed as the sum of the sub-areas of interest (2 items), lifestyle (5 items), diet (4 items), and prevention (3 items). Since each subarea has a different total number of questions, only the tendency is expressed in the graph.

#### Correlations between oral health behaviors and oral health literacy

There was a significant positive correlation between oral health literacy and oral health behaviors (*r* = 0.526, *P* < 0.001, Table 4).

**Table 4:** Correlations between oral health behaviors and oral health literacy

	Verbal oral health literacy	Functional oral health literacy	Oral health literacy	Oral health behaviors
Verbal oral health literacy	1			
Functional oral health literacy	0.327^**^	1		
Oral health literacy	0.789^***^	0.838^***^	1	
Oral health behaviors	0.353^**^	0.497^***^	0.526^***^	1

^***^P < 0.001, ^**^P < 0.05 by Pearson’s correlation test

#### Association between oral health literacy and oral health behaviors

Functional oral health literacy and oral health literacy were significantly associated with higher oral health behaviors (Beta = 0.20 (95% CI 0.01–0.43) and Beta = 0.26 (95% CI 0.04–0.33), respectively), but verbal oral health literacy was not (Beta = 0.126, *P =* 0.151). Furthermore, a poor interest in perceived oral health status was significantly associated with lower oral health behavior (Table 5).

**Table 5:** Multivariable linear regression analysis investigating oral health behavior as a function of oral health literacy, which comprises verbal and functional oral health literacy

	Oral health behaviors			
	^a^Model 1		^b^Model 2	
	Beta (95%CI)	*P*	Beta (95%CI)	*P*
Verbal oral health literacy	0.13 (− 0.06; 0.37)	0.15		
Functional oral health literacy	0.20 (0.01; 0.43)	0.04^*^		
Oral health literacy (Verbal oral health literacy + Functional oral health literacy)			0.26 (0.04; 0.33)	0.01^*^
Sex (Ref. Male)				
Female	0.03 (− 2.96; 4.23)	0.73	0.03 (− 2.94; 4.21)	0.73
Age (Ref. 19–39)				
40–59	− 0.08 (−5.09; 2.20)	0.43	− 0.08 (− 5.07; 2.18)	0.43
60–80	0.11 (− 2.76; 9.18)	0.29	0.11 (− 2.85; 8.86)	0.31
Education (Ref. ≤ Primary school)				
Middle school	0.17 (− 0.92; 9.52)	0.11	0.17 (− 0.87; 9.53)	0.10
High school	0.10 (− 2.90; 6.79)	0.43	0.10 (− 2.89; 6.76)	0.43
≥College	0.04 (−4.56; 6.67)	0.71	0.05 (− 4.49; 6.68)	0.70
Income [Units: 10,000 KRW] (Ref. ≤ 99)				
100–199	0.00 (−3.64; 3.68)	0.99	−0.00 (− 3.66; 3.62)	0.99
≥200	0.14 (− 1.52; 9.03)	0.16	0.14 (− 1.49; 9.02)	0.16
Occupation (Ref. Yes)				
No	0.06 (−2.21; 4.65)	0.48	0.06 (−2.21; 4.62)	0.49
Marital status (Ref. Single)				
Married	−0.01 (−3.80; 3.47)	0.93	− 0.01 (− 3.73; 3.49)	0.95
Years living in South Korea (Ref. < 7 years)				
≥7 years	− 0.11 (−5.14; 1.11)	0.20	− 0.11 (− 5.13; 1.09)	0.20
Number of diseases (Ref. 0)				
1	−0.05 (− 4.81; 2.65)	0.57	− 0.05 (− 4.80; 2.63)	0.56
2	− 0.09 (−6.95; 2.12)	0.29	− 0.09 (− 6.93; 2.10)	0.29
≤ 3	− 0.11 (−7.40; 1.92)	0.25	− 0.11 (− 7.29; 1.96)	0.26
Private insurance (Ref. Yes)				
No	− 0.10 (− 4.94; 1.18)	0.23	−0.10 (− 4.98; 1.01)	0.19
Perceived oral health status (Ref. Good)				
Normal	−0.15 (−8.03; 2.09)	0.25	− 0.15 (− 7.85; 2.15)	0.26
Poor	− 0.19 (− 8.80; 1.62)	0.18	− 0.18 (− 8.55; 1.70)	0.19
Interest in perceived oral health status (Ref. Good)				
Normal	− 0.09 (− 5.11; 1.41)	0.26	− 0.09 (− 5.13; 1.35)	0.25
Poor	− 0.26 (− 12.44; − 1.29)	0.02^*^	−0.27 (− 12.51; − 1.50)	0.01^*^
Tooth brushing (Ref. 1)				
2	0.24 (− 0.67; 10.24)	0.09	0.25 (− 0.32; 10.32)	0.07
≤ 3	0.18 (−2.09; 9.02)	0.22	0.18 (−1.97; 9.06)	0.21
Use of dental products (Ref. 1)				
2	0.11 (− 4.31; 8.49)	0.52	0.11 (−4.28; 8.47)	0.52
≤ 3	0.31 (− 0.80; 13.01)	0.08	0.32 (− 0.63; 13.07)	0.08
Dental care utilization (Ref. Yes)				
No	− 0.15 (− 5.87; 0.17)	0.06	− 0.15 (− 5.83; 0.18)	0.07

The results were derived using the standardized regression coefficient—beta—to compare the magnitudes of the impact of oral health literacy (composed of verbal and functional oral health literacy), verbal oral health literacy, and functional oral health literacy

*Abbreviations*: *CI* confidence interval, *Ref* reference group

^*^P < 0.05

^a^Model 1 and ^b^Model 2 adjusted for sex, age, education, income, occupation, marital status, years living in South Korea, number of diseases, private insurance membership, perceived oral health status, interest in perceived oral health status, tooth brushing, use of dental products, and dental care utilization

^a^Model 1 identifies the effects of verbal oral health literacy and functional oral health literacy after the confounding variables have been adjusted for

^b^Model 2 identifies the effects of oral health literacy itself, which combines verbal oral health literacy and functional oral health literacy, after the confounding variables have been adjusted for

## Discussion

The present study aimed to investigate North Korean defectors’ comprehension of oral health information by measuring their oral health literacy and investigating its association with oral health behaviors. The results showed that the mean oral health literacy score was 44 out of a maximum possible score of 72, with a mean verbal oral health literacy score of 14 (maximum 30) and mean functional oral health literacy score of 30 (maximum 42).

Khan et al. [21] reported a mean verbal oral health literacy score of 22.98 ± 5.1 among 150 adults aged 50 years or older who visited a dental clinic for treatment, measured using the REALD-30 scale. In Kim’s [13] study on foreigners living in Korea, the mean verbal oral health literacy score was 6.2 ± 6.7. North Korean defectors, the subjects of this study, had a mean verbal oral health literacy score (14.10 ± 7.79) between native citizens and immigrants with a different first language. This indirectly shows that North Korean defectors have limitations in oral health literacy despite the fact that they use a language similar to that used by South Koreans. Hence, specific and customized communication methods are needed to improve the oral health of North Korean defectors.

The positive association between oral health literacy and oral health behaviors, confirmed in this study, has been documented in multiple previous studies. Ueno et al. [22] reported that people with higher oral health literacy brushed their teeth or dentures more frequently, checked their oral condition using a mirror, sought regular dental checkups, and had better oral hygiene. Furthermore, they had more remaining natural teeth and fewer decayed teeth. In contrast, participants with low oral health literacy had a poorer periodontal status, based on the community periodontal index. The oral health literacy instrument used in this study was developed for English speaking participants and was adapted to the Japanese-speaking participants. However, as this tool did not modify all areas, it was difficult to generalize the research results. Naghibi Sistani et al. [23] examined the effects of oral health literacy on self-reported oral health status (good vs poor), and found that poor tooth brushing behavior (odds ratio = 3.35), and low oral health literacy scores (odds ratio = 1.58) were significant risk indicators of poor self-reported oral health. Although this study raised concerns about the response bias of self-assessment questions, it stressed that oral health literacy should be a priority in oral health promotion programs as a determinant of oral health. Wehmeyer et al. [24] investigated the association between oral health literacy (measured with the REALD-30 scale) and periodontal health status (assessed with a clinical periodontal examination) among patients with periodontal disease. Despite the fact that the subjects were highly educated, more than one-third had low oral health literacy. Approximately 53% of the subjects had severe periodontitis, 29% had moderate periodontitis, and 18% had mild or no periodontitis. Low oral health literacy was significantly associated with severe periodontal disease (*P* < 0.001), and the probability of worsening periodontal disease increased by 1.19 times with every one-unit reduction of oral health literacy (*P* = 0.002). Subject selection in this study had a limitation, it was designed to only include patients who visited a dental clinic at a university. However, the results of this study have many positive aspects that could be useful in suggesting effective and diverse communication and education methods to improve oral health literacy. Lee et al. [25] examined the association between oral health literacy and oral health status, and the role of self-efficacy. Higher oral health literacy was significantly associated with a better oral health status, and self-efficacy mediated this relationship. Although the latter was a prospective cohort study, only subjects enrolled in North Carolina’s Special Supplemental Nutrition Program for Women, Infants, and Children (WIC) were included. As such, it is difficult to generalize the results because male subjects were absent. Holtzman et al. [26] investigated the association between oral health literacy, measured using the Rapid Estimate of Adult Literacy in Medicine and Dentistry, and failing to show for dental appointments. A greater percentage of people with high oral health literacy (54.2%) obtained health information via the internet compared to people with low oral health literacy (28.1%). They predicted that people with poor health literacy may also have poor oral health literacy. This was attributed to a restricted ability to access health information due to financial status and time limitations, as well as difficulties understanding health information (e.g., on the internet) and attending dental appointments. Some studies also reported that oral health literacy measured using REALD-30 was not significantly associated with the use of dental care [27], while other studies reported that oral health literacy was significantly associated with perceived dental health status [28]. Taken together, these studies confirm, from various perspectives, that oral health literacy is significantly associated with higher oral health behaviors. This implies the importance of developing effective educational materials and intervention programs, tailored to the oral health literacy of the recipients, to improve oral health [22, 23]. Studies have also suggested that both written and verbal communication can improve oral health literacy [21, 26].

In this study, we found that while verbal oral health literacy was not significantly associated with oral health behaviors, functional oral health literacy did show a significant association. This suggests that North Korean defectors have limitations in performing oral health behaviors simply by being exposed to oral health information. In order to engage in oral health behaviors, they must first achieve functional oral health literacy by being able to absorb and understand information. Fragmentary knowledge obtained as a result of education, or through the media while staying in South Korea, cannot by itself lead to the actual performance of such behaviors. Park [29] reported that “educational effects” have an impact on the health literacy of North Korean defectors, and this finding is in agreement with the positive association of oral health literacy and oral health behaviors with educational level found in this study (*p* < 0.001 for one-way ANOVA test, not shown in the Table). Therefore, improving oral health behaviors in North Korean defectors through educational support would have a positive impact on their settlement in South Korea.

To improve oral health behaviors among North Korean defectors, a customized approach should be taken, specifically to improve the oral health literacy of those defectors who lack understanding of the South Korean healthcare system, and those maintaining inappropriate healthcare behaviors. Thus, the provision of oral health information should facilitate improved oral health behaviors, which should in turn result in improved clinical oral health. In addition, the results of this study showed that oral health behavior was negatively associated with poor interest in oral health. This suggests that if oral health literacy is improved through systematic oral health education, interest in oral health can be increased. Brushing teeth, cleaning dentures frequently, self-examination in a mirror, regular dental check-ups, and maintaining better oral hygiene [22] are examples of expressions of interest, which may ultimately have a positive effect on oral health behavior.

The present study has a few limitations. First, we randomly selected North Korean defectors in the metropolitan area, and the higher proportion of women limited the generalizability of the findings to the entire North Korean defector population. However, as shown in the entry data of North Korean defectors, women outnumber men threefold [1]. Therefore, the composition of our sample appears to be representative of the true population. Second, self-reported questionnaires are vulnerable to self-reporting bias (e.g., response bias). Third, this study adopted a cross-sectional design and simply examined the association between oral health literacy and oral health behaviors. As a result, we could not examine the causality relationships between the two variables. Lastly, we were unable to assess whether the lower level of oral health literacy among the North Korean defector population was indeed different compared to native South Koreans living in the same region, due to the lack of an adequate matched control group. Nevertheless, this study is meaningful in that it sheds light on the association between oral health literacy and oral health behaviors among North Korean defectors in South Korea, although they face a smaller language barrier than that of other immigrants. In the future, it will be necessary to develop instruments to measure oral health literacy, as well as culturally appropriate educational interventions to promote North Korean defectors’ health. The results of this study serve as an evidence base to inform future studies, in terms of the analysis of oral health literacy, with the overarching aim of facilitating the development of health policies targeted not only towards North Korean defectors, but also other immigrant populations as well.

## Conclusion

This study confirmed that the oral health literacy of North Korean defectors was significantly associated with higher oral health behaviors. These findings suggest that instruments for evaluating oral health literacy, as well as educational interventions, are required for the improvement of oral health among North Korean defectors. In the future, we will confirm the consistency of education through large-scale cohort studies. Through the provision of optimal oral health, we expect to improve quality of life and facilitate the stable settlement of North Korean defectors in South Korea.

## Data Availability

Datasets for the current study are not publicly available to protect the anonymity of the respondents.

## Abbreviations

OHLI: Oral Health Literacy Instrument
OHLI-K: Oral Health Literacy Instrument in Korea
REALD: Rapid Estimate of Adult Literacy in Dentistry
TOFHLiD: Test of Functional Health Literacy in Dentistry
TOKFHLiD: Korean Functional Health Literacy in Dentistry
